# Synthesis of Porous MgAl-LDH on a Micelle Template and Its Application for Efficient Treatment of Oilfield Wastewater

**DOI:** 10.3390/molecules28186638

**Published:** 2023-09-15

**Authors:** Bingbing Bai, Qingchen Wang, Yan Sun, Rui Zhou, Gang Chen, Ying Tang

**Affiliations:** 1 Shaanxi Province Key Laboratory of Environmental Pollution Control and Reservoir Protection Technology of Oilfields, Xi’an Shiyou University, Xi’an 710065, China; 21211070906@stumail.xsyu.edu.cn (B.B.); ruizhou@xsyu.edu.cn (R.Z.); gangchen@xsyu.edu.cn (G.C.); 2 Shaanxi University Engineering Research Center of Oil and Gas Field Chemistry, Xi’an Shiyou University, Xi’an 710065, China; 3 Changqing Drilling Company of CCDC, Xi’an 710060, China; zjwqch@cnpc.com.cn (Q.W.); zjs3sy@cnpc.com.cn (Y.S.)

**Keywords:** porous LDH, sulfonated lignite, adsorption reaction

## Abstract

In this paper, a series of porous hierarchical Mg/Al layered double hydroxides (named as LDH, TTAC-MgAl-LDH, CTAC-MgAl-LDH, and OTAC-MgAl-LDH) was synthesized by a simple green hydrothermal method using wormlike micelles formed by salicylic acid and surfactants with different carbon chain lengths (0, 14, 16, and 18) as soft templates. BET, XRD, FTIR, TG, and SEM characterizations were carried out in order to investigate the structure and properties of the prepared materials. The results showed that the porous hierarchical CTAC-MgAl-LDH had a large specific surface area and multiple pore size distributions which could effectively increase the reaction area and allow better absorption capability. Benefiting from the unique architecture, CTAC-MgAl-LDH exhibited a large adsorption capacity for sulfonated lignite (231.70 mg/g) at 25 °C and a pH of 7, which outperformed the traditional LDH (86.05 mg/g), TTAC-MgAl-LDH (108.15 mg/g), and OTAC-MgAl-LDH (110.51 mg/g). The adsorption process of sulfonated lignite followed the pseudo-second-order kinetics model and conformed the Freundlich isotherm model with spontaneous heat absorption, which revealed that electrostatic adsorption and ion exchange were the main mechanisms of action for the adsorption. In addition, CTAC-MgAl-LDH showed a satisfactory long-time stability and its adsorption capacities were still as high as 198.64 mg/g after two adsorption cycles.

## 1. Introduction

Sulfonated lignite (SL) is produced by introducing sulfonic acid group SO_3_H into condensed aromatic rings and aliphatic side chains of coal following sulfonation with concentrated sulfuric acid [1,2]. As its low viscosity and great dispersibility, SL is employed in petroleum exploration as a filtrate reduction, viscosity reducer, and pressure-reducing agent [3,4,5]. During the oil recovery process of deep shale, however, a large amount of highly concentrated and difficult-to-degrade drilling fluid wastewater containing sulfonated lignite macromolecules will be generated, causing deterioration of ecosystems as well as water quality and easily reacting with chlorine used in water treatment to produce carcinogens [6,7,8,9,10,11]. There are many methods to treat drilling wastewater, such as Fenton oxidation, the gravity separation method, the membrane separation method, the flocculation method, the salting-out method, the activated sludge method, the biological filter method, and peroxydisulfate oxidation [12,13,14,15,16,17,18,19,20]. However, most of them have shortcomings such as non-renewable raw materials, high operating costs, and incomplete treatment of pollutants. Therefore, finding an effective method to treat SL in drilling fluid wastewater is crucial to the protection of water resources.

Layered double hydroxides (LDHs) are anionic clay minerals with thermal stability, ion exchange properties, memory effects, and acid–base dual properties [21,22,23]. Yao et al. used G-LDH prepared by the hydrothermal method to achieve efficient removal of MO from wastewater by ion exchange [24]. Despite showing satisfactory results in the field of water treatment, traditional LDHs are prone to agglomeration after calcination under high temperatures, leading to their poor dispersion and consequently poor performance in removing pollutions in oilfield wastewater. To solve the above problems, the preparation of LDHs with porous structures is highly desirable. Different chemical and physicochemical methods have been employed to prepare diversified porous adsorption materials. The template synthesis, which uses either hard templates or soft templates, is the most commonly used method [25,26,27]. However, hard template requires a multistep synthetic process and gives low-product yield [28]. Utilizing soft template is promising due to its simple and effective control of the morphology and size of the synthesized material by the choice of template agent [29]. Studies have shown that surfactants and organic counterions (such as salicylic acid) usually self-assemble into a series of wormlike micelles, which built a delicate large porous structure so as to give great potential to prepare ordered porous materials [30,31].

In this work, porous MgAl-LDH was prepared under a hydrothermal condition by employing worm-like micelles derived from trimethyl ammonium chloride with different carbon chain lengths (tetradecyl trimethyl ammonium chloride, cetyl trimethyl ammonium chloride, and octadecyl trimethyl ammonium chloride). Highly efficient adsorption performance was expected over this porous material and adsorbent conditions, parameters that may affect the adsorption, including pH, and the adsorbent dosage were evaluated. Both kinetic and equilibrium isotherm models were applied to establish the rate of adsorption and the adsorption capacity. Furthermore, the comparative characterizations of CTAC-MgAl-LDH before and after adsorption were well conducted to explore the corresponding adsorption mechanism towards SL.

## 2. Results and Discussion

### 2.1. Structural Characterization of Porous MgAl-LDH

**Figure 1:**
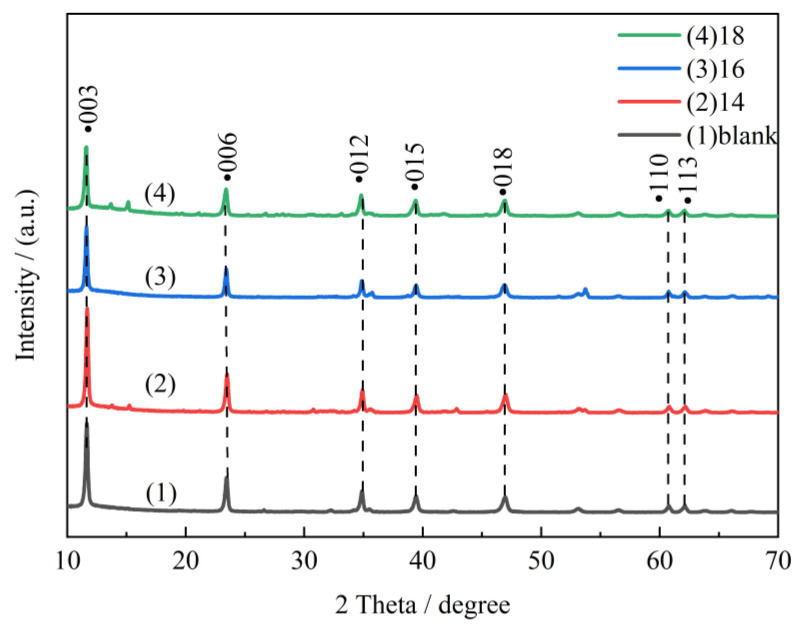
XRD patterns of LDH at different alkyl carbon chain lengths.

Figure 1 shows XRD patterns of the sample synthesized by a 3:1 mole ratio of Mg/Al at 160 °C for 6 h. The 2θ peaks located at 11.60°, 23.50°, 34.90°, 39.50°, 47.00°, 60.90°, and 62.10° are ascribed to the diffractions of basal planes of (003), (006), (012), (015), (110), (018), and (113) of LDH materials [32]. It can be indicated that the surfactants with different carbon chain lengths can all induce the orderly deposition of metal salt solutions, resulting in the preparation of LDH materials with good and layered structures. In addition, CTAC-MgAl-LDH, with a carbon chain length of 16, showed the lowest diffraction peak at (006), (012), (015), and (118) compared to other MgAl-LDHs, indicating that the micelle-templated hydrotalcite prepared with CTAC as surfactant had the best dispersion, which was favorable to enhance the adsorption activity of CTAC-MgAl-LDH to SL. This observation is consistent with the superior adsorption performance exhibited by CTAC-MgAl-LDH with a carbon chain length of 16, as mentioned later in the text.

**Figure 2:**
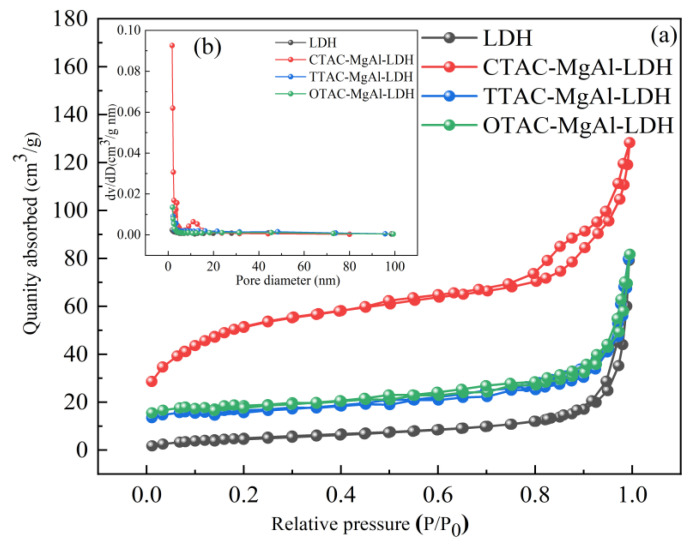
N_2_ adsorption–desorption isotherms (**a**) and pore size distribution curve (**b**) of LDH.

The surface properties (such as specific surface area and pore-size distribution) are important factors of adsorbent; N_2_-adsorption/desorption measurements (Figure 2a) are performed over traditional hydrotalcite (LDH) and templated hydrotalcite. All samples displayed a type IV isotherm with H4 hysteresis loops and a type IV isotherm with H3 hysteresis loops, indicating the presence of mesopores. In addition, CTAC-MgAl-LDH had a large hysteresis loop and showed a significant adsorption slope at higher adsorption pressures (*p* > 0.8), which was attributed to the improved adsorption–desorption capacity of the material due to the macroporous structure. This is further verified by the pore size distribution curve (Figure 2b). As can be seen from the graph, CTAC-MgAl-LDH exhibited a wide size distribution (5–80 nm) and was mainly concentrated between 5 and 20 nm, indicating that the sample was composed of hierarchical porous materials with mesopores and macropores [33].

The BET surface area, pore volume, and pore diameter of the relative samples determined via N_2_-adsorption/desorption measurements are listed in Table 1. Compared to LDH, the introduction of a micelle template significantly increased the specific surface area. The enhancement was particularly prominent in CTAB-MgAl-LDH, with an increase from 19.21 to 174.24 m^2^/g. An increase in pore size was observed for CTAC-MgAl-LDH from 2.42 to 4.72 nm. In addition, the macropore volume of CTAC-MgAl-LDH was found to be increased to 0.20 cm^3^/g which indicates that the macropores structure after adding micelle template provided a high adsorption potential for SL.

**Figure 3:**
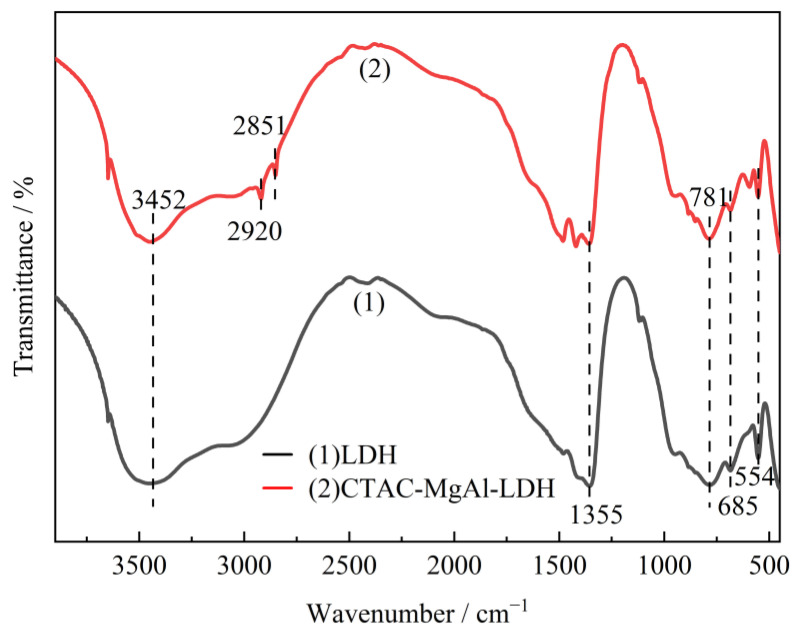
FTIR spectra of MgAl-LDH prepared by different methods.

FTIR spectra of samples were performed to analyze the chemical structure. Figure 3 presents the FTIR spectra of LDH and CTAC-MgAl-LDH. The broad and strong absorption peak observed at 3452 cm^−1^ was assigned to the stretching vibration of the O-H groups in the hydroxide layer and the water molecules. The absorption bands at 1355 cm^−1^ were attributed to the asymmetric stretching vibration of C-O, indicating the existence of the carbonate anion in the MgAl-LDH [34]. And the absorption peaks at 500 cm^−1^ to 900 cm^−1^ are related to metal–oxygen and metal–hydroxyl vibrations in the hydrotalcite lattice. Compared with LDH spectra, CTAC-MgAl-LDH showed two small and narrow peaks at 2920 and 2851 cm^−1^, which were attributed to CH_3_ asymmetric and CH_2_-symmetric stretching vibrations carried by the surfactant, indicating a good interaction between micelles and hydrotalcite [35].

**Figure 4:**
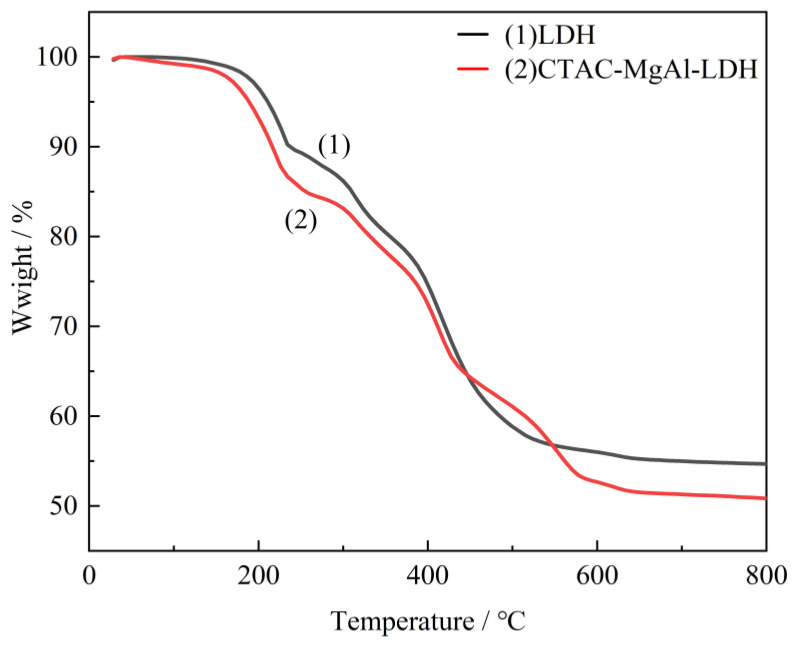
TG curves of LDH and CTAC-MgAl-LDH.

Thermal analysis experiments were employed to characterize the thermal stability of the as-prepared LDH and CTAC-MgAl-LDH. It can be observed from the Figure 4 that two weight loss steps both exist in LDH and CTAC-MgAl-LDH. The first step from room temperature to approximately 240 °C corresponded to the removal of physisorbed water, interlayer water, and the dehydroxylation of the LDH layers. The second weight loss step in the temperature range from 240 to 600 °C was due to the dehydroxylation of the hydrotalcite layers accompanied by the decomposition of interlayer carbonate anions [36]. The micellar template weakens the interaction of interlayer anions with the laminate, making it easier to achieve the mutual exchange of anions. This is illustrated by the higher weight loss of the CTAC-MgAl-LDH carbonate anion (47.15%) compared to LDH (43.67%) at 600 °C.

### 2.2. Adsorption Performance

**Figure 5:**
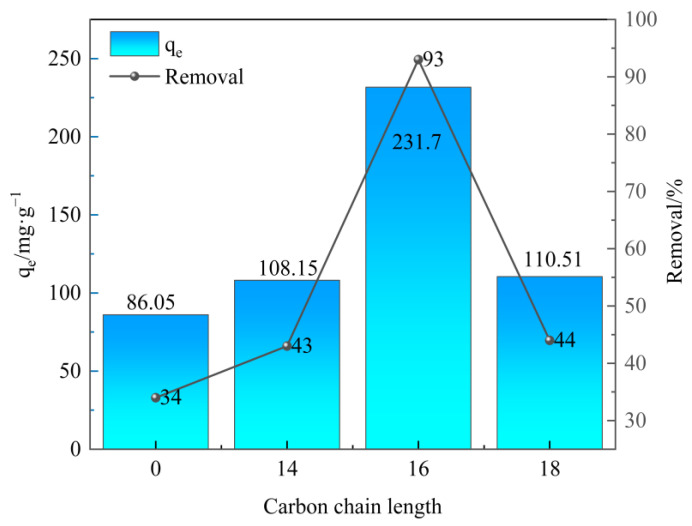
Effect of carbon chain length on sulfonated lignite adsorption.

Sulfonated lignite was chosen as the target contaminant to study the adsorption behavior of as-prepared samples. Under the conditions of 25 °C, an initial pH of 7, an adsorbent dosage of 0.40 g/L, and a sulfonated lignite initial concentration of 100 mg/L, the adsorption performance of adsorbent prepared by surfactants with different carbon chain lengths is shown in Figure 5. From the result, it can be seen that the prepared hydrotalcite derived from the micellar template had a significantly better removal efficiency of sulfonated lignite with increasing the carbon chain length from 0 to 16. The best removal efficiency was found over 16 carbon chain lengths with the adsorption capacities of 231.70 mg/g. However, when the carbon chain length of surfactant with 18 was introduced, the adsorption capacity and removal rate decreased to 110.51 mg/g and 44%, respectively. It should be due to the increasing viscosity of the surfactant with increasing carbon chain length, causing the agglomeration of OTAC-MgAl-LDH and poor dispersion of samples suggested as the result of XRD [37].

**Figure 6:**
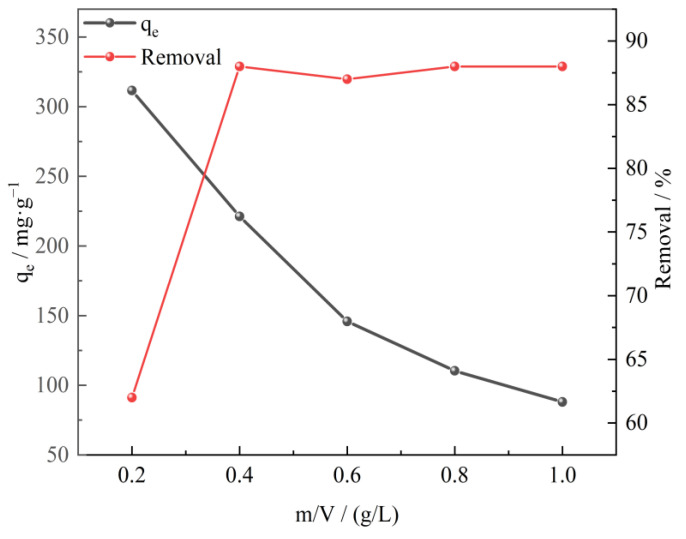
The effect of adsorbent dosage on SL adsorption for CTAC-MgAl-LDH.

The adsorbent dosage is of great significance to the adsorption process of sulfonated lignite. In the research, the effect of adsorbent dosage was analyzed by performing the adsorption tests with varying amounts (0.20 to 1.00 g/L) of adsorbent at an initial SL concentration of 100 mg/L, a reaction temperature of 25 °C, and an initial pH of 7 of the solution. It can be seen from Figure 6 that as the amount of adsorbent increased, the equilibrium adsorption capacity obviously decreased with removal efficiency and reached a maximum value of 88.4% when the adsorbent dosage was 0.40 g/L. This trend was expected because higher dosages of adsorbent might cause greater availability of surface area and exchangeable binding sites. However, it was found that the removal rate was basically unchanged, even after increasing the amount of adsorbent due to the saturation adsorption of CTAC-MgAl-LDH [38].

**Figure 7:**
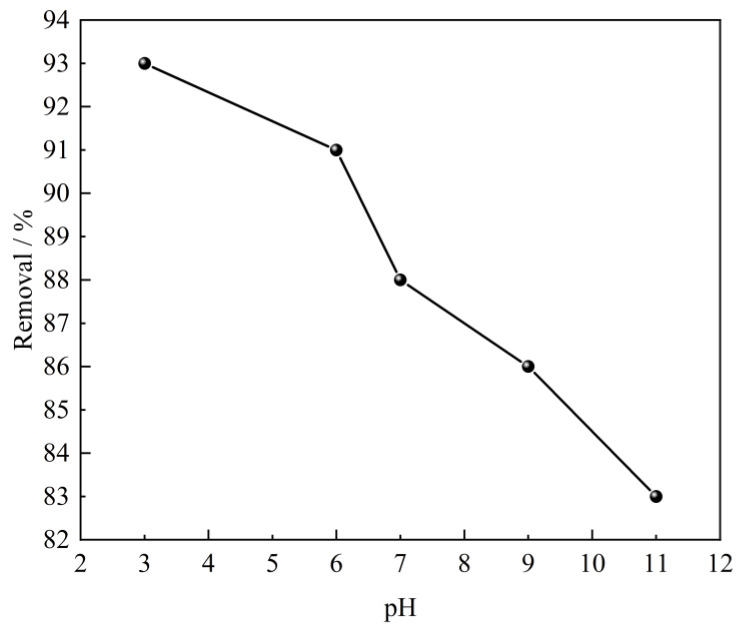
Effect of initial solution pH on SL adsorption using CTAC-MgAl-LDH.

The pH of the solution is acknowledged as one of the important factors that affect the adsorption performance of the adsorbent. It may considerably affect the degree of ionization, surface charge, and protonation of the functional groups of the adsorbent [39]. Therefore, the effect of solution pH on SL adsorption performance over CTAC-MgAl-LDH was evaluated with an initial pH ranging from 3 to 11 at an initial SL concentration of 100 mg/L, an adsorbent dosage of 0.40 g/L, and a temperature of 25 °C. It is evident from Figure 7 that the adsorption of SL by CTAC-MgAl-LDH occurs more easily under acidic conditions because a large amount of H^+^ is attached to the surface of hydrotalcite under acidic conditions and it is easy to generate electrostatic gravitational force with the negatively charged sulfonic acid ions to promote the adsorption. Furthermore, the decrease in removal cannot be avoided when the pH of solution increased up to 11 due to a large amount of negatively charged OH^−^ and sulfonic acid ions giving a competitive adsorption effect [40].

### 2.3. Adsorption Kinetics Study

**Figure 8:**
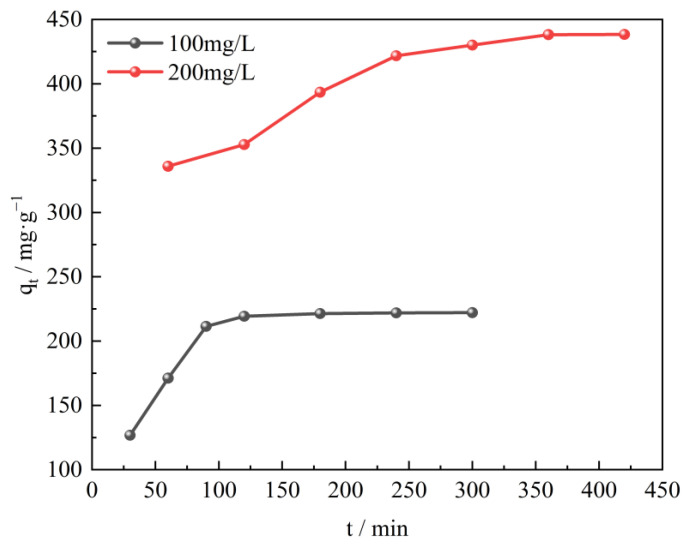
Effect of contact time on SL adsorption using CTAC-MgAl-LDH for initial SL concentrations of 100 and 200 mg/L.

The adsorption kinetics illustrates the pollutants adsorption rate and eventually explores the mechanism of adsorption and the rate limiting steps involved. The effects of contact time of CTAC-MgAl-LDH and SL on the adsorption property were investigated by performing experiments at different SL concentrations (100 and 200 mg/L) and varying contact times at 25 °C and pH 7. As shown in Figure 8, at low initial concentration (100 mg/L), the adsorption capacity can reach up to 350 mg/L during the first 90 min due to the abundance of adsorption sites on the adsorbent surface. After a period of time, the remaining empty adsorption sites are difficult to be occupied due to the repulsive forces between the sulfonated lignite molecules and the bulk phase [41], resulting in a low adsorption rate until equilibrium is reached. Different adsorption time was required to reach equilibrium at different initial SL concentrations. The equilibrium times of 120 and 350 min corresponded to the initial concentrations of 100 and 200 mg/L, which indicates that the higher SL concentration, the longer time needed to reach equilibrium [42].

To investigate the adsorption process of SL on CTAC-MgAl-LDH, the pseudo-first-order, pseudo-second-order, intraparticle diffusion, and liquid film diffusion models were used to fit the experimental results and the corresponding equations are specified as follows:

(1) $$\ln\left(q_{e}-q_{t}\right)=\ln q_{e}-k_{1}t$$

(2) $$\frac{t}{q_{t}}=\frac{1}{k_{2}q_{e}{}^{2}}+\frac{1}{q_{e}}t$$

(3) qt=kpt12+C

(4) $$\ln\left(1-\frac{q_{t}}{q_{e}}\right)=-K_{fd}t$$

In the given equations, *k*_1_ and *k*_2_ represent the rate constants (min^−1^ and g/(mg·min), respectively) for the respective kinetic models. The constant *k_p_* corresponds to the rate of ion diffusion (mg/(g·min^0.5^)) while *C* is a constant related to the thickness of the boundary layer (mg/g). Additionally, *K_fd_* denotes the rate constant for liquid film diffusion (h^−1^).

**Figure 9:**
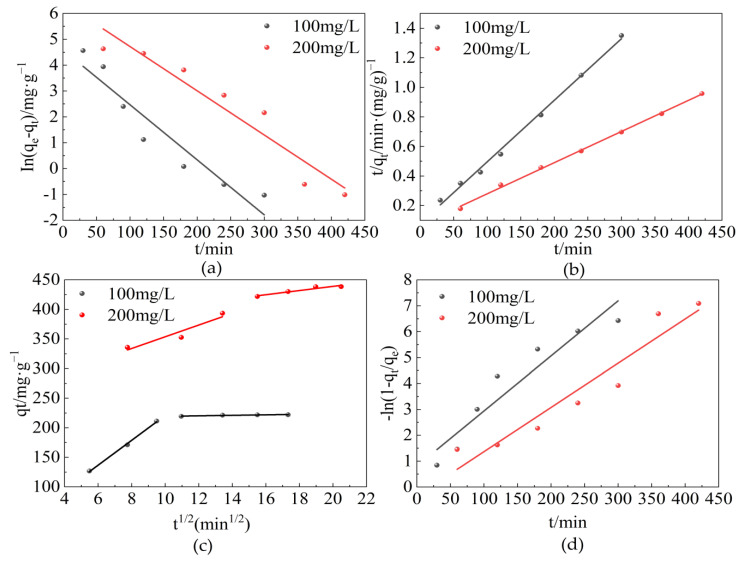
The four kinetic models for the adsorption of SL by CTAC-MgAl-LDH: (**a**) pseudo-first-order model, (**b**) pseudo-second-order model, (**c**) particle internal diffusion, and (**d**) liquid film diffusion.

The fitting isotherm and kinetic parameters obtained by the linear regression are shown in Figure 9 and Table 2, respectively. From the results, it can be seen that the correlation coefficient (*R*^2^) obtained from the pseudo-second-order kinetic model (*R*^2^ was in the range of 0.996–0.998) was higher than that obtained from the pseudo-first-order kinetic model (*R*^2^ was in the range of 0.906–0.917). Furthermore, the equilibrium adsorption values calculated by the second order model (*q_e,cal_*) matched well with experimental adsorption results (*q_e,exp_*), which further confirmed that the kinetics of adsorption by CTAC-MgAl-LDH for the SL was best described by the pseudo-second-order model, which indicated that the rate controlling mechanism for adsorption was chemisorption caused by the electron exchange or sharing between the adsorbate and adsorbent [43]. In addition, it was found that the linear fitting correlation coefficient of the intra-particle diffusion model could reach up to 0.998 while the linear fitting correlation coefficient of the liquid diffusion model was between 0.901 and 0.906, thus indicating that the intra-particle diffusion model was the main rate control step of adsorption.

### 2.4. Adsorption Isotherm

Adsorption isotherm studies usually describe the equilibrium adsorption behavior at constant temperatures [44]. This study analyzes the experimental data by employing the Langmuir, Freundlich, and Dubinin–Redushckevich models. The fitting relationships of these models can be observed in Equations (5)–(7), respectively.

(5) $$\frac{c_{e}}{q_{e}}=\frac{c_{e}}{q_{m}}+\frac{1}{b}q_{m}$$

(6) $$\mathrm{lg}q_{e}=\mathrm{lg}K_{f}+\frac{1}{n}\mathrm{lg}c_{e}$$

(7) lnqe=lnqmax−βε2ε=RTln(1+1ce)E=(12β)12

where *b* is a constant, *K_f_* is associated with the relationship between water hydrotalcite and sulfonated lignite, *n* is a constant related to the adsorption strength, and when *n* > 1, the adsorption reaction is more favorable. *β* is the activity constant (mol^2^/kJ^2^), *ε* represents the Polanyi potential, and *E* corresponds to the average free energy (kJ/mol).

**Figure 10:**
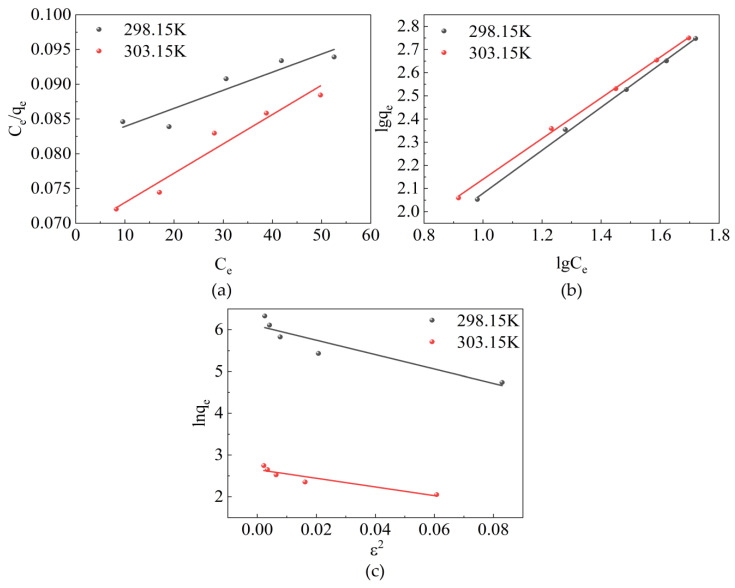
Adsorption isothermal models for the adsorption of SL by CTAC-MgAl-LDH: (**a**) Langmuir model, (**b**) Freundlich model, and (**c**) Dubinin–Radushkevich (D–R) model.

The aforementioned isothermal models fitted based on experimental data are shown in Figure 10 and the corresponding adsorption isothermal parameters are shown in Table 3. *R*^2^, obtained by the Freundlich isotherm model, is greater than 0.998 and that by the Langmuir adsorption isotherm model is between 0.879–0.952. These values indicate that the adsorption process of the adsorbent conforms to the Freundlich isotherm model. Therefore, the adsorption process was dominated by reversible adsorption with a different affinity and belonged to multilayer adsorption [45]. The values of *n* (1.08 and 1.14 at 298.15 and 303.15 K) for the best-fit Freundlich model were greater than 1, indicating that the adsorption process of sulfonated lignite proceeded easily. The values of activation energy calculated in the D–R model were 170.20 and 219.00 J/mol at 298.15 K and 303.15 K, which are both less than 8 kJ/mol, indicating that electrostatic gravity is the main force in the adsorption process [46].

### 2.5. Thermodynamic Study of Adsorption

**Figure 11:**
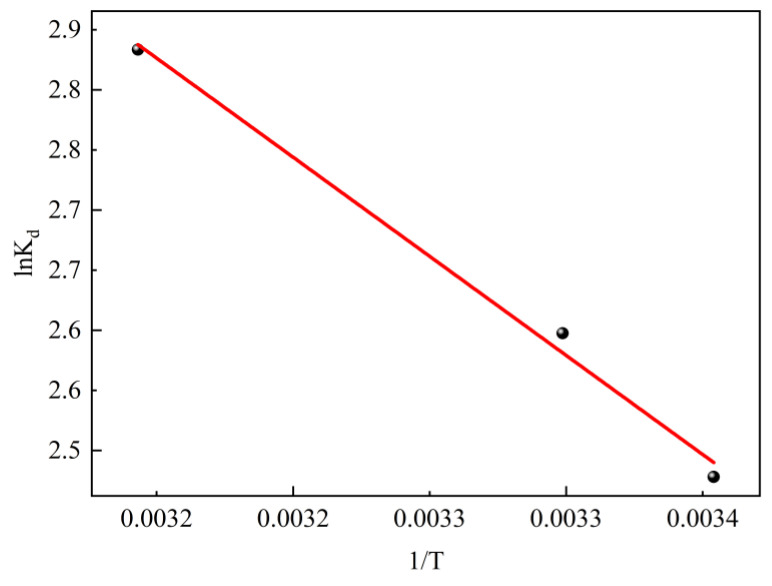
Adsorption thermodynamics of SL on CTAC-MgAl-LDH.

To confirm the nature of the adsorption process, experimental data for SL adsorption under equilibrium at different temperatures were used to evaluate the thermodynamic parameters. The fitting isotherms are shown in Figure 11 and the calculated parameters are summarized in Table 4. It can be found that the standard Gibbs free energy change value (Δ*G*) at different temperatures is negative, indicating that the adsorption process can be carried out spontaneously. The positive value of standard enthalpy change (Δ*H*) can demonstrate that the adsorption process is an endothermic process. Therefore, an appropriate temperature increase is beneficial for promoting the adsorption of SL by CTAC-MgAl-LDH, which was consistent with the experimental results of temperature effects. Simultaneously, the positive value of standard entropy change (Δ*S*) of adsorption reflects that the process of adsorption is an entropy-increasing process [47].

### 2.6. Regeneration of Adsorbent

**Figure 12:**
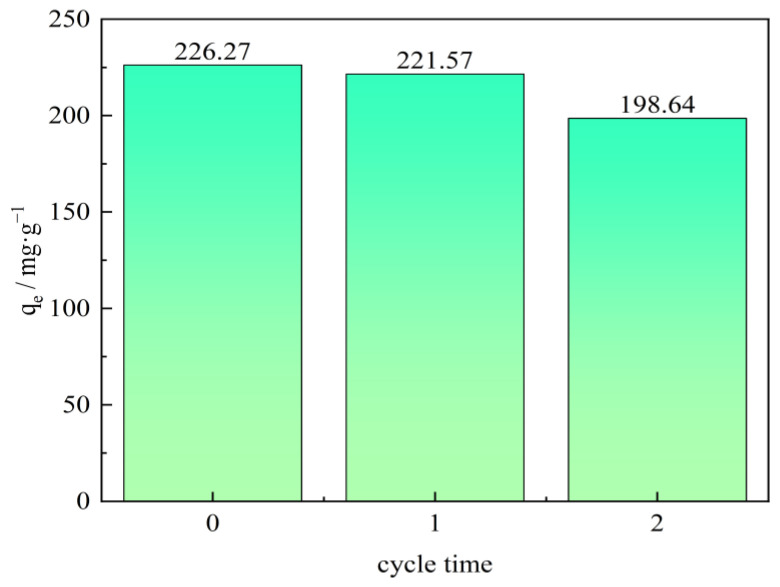
Regeneration performance of SL by CTAC-MgAl-LDH.

To be an economical and effective adsorbent in the oilfield wastewater treatment process, an adsorbent that could be easily regenerated and reused is crucially important and extremely welcome [48]. Adsorbent regeneration is the process of reproducing the adsorbent that has been used in the adsorption process. In the experiment, a NaOH solution was used to study the regeneration of the CTAC-MgAl-LDH. At room temperature, the CTAC-MgAl-LDH with saturated adsorption was immersed in an aqueous solution with a pH of 13 and stirred for 12 h to achieve the desorption process, which was the adsorbent for primary regeneration after repeated washing. The regeneration performance of the CTAC-MgAl-LDH was investigated when the initial concentration of the sulfonated lignite was 200 mg/L at 25 °C and a pH of 7. It can be seen from the result of Figure 12 that after two cycles of regeneration, the adsorption capacity of the hierarchical MgAl-LDH on SL slightly decreased from 226.27 to 198.64 mg/g, reflecting the potential for practicality of prepared CTAC-MgAl-LDH. The decrease in adsorption affinity can be attributed to the loss of adsorbent during the adsorption cycle and the incomplete desorption of some adsorption sites of SL on the CTAC-MgAl-LDH surface.

### 2.7. Characterization of CTAC-MgAl-LDH before and after Adsorption

**Figure 13:**
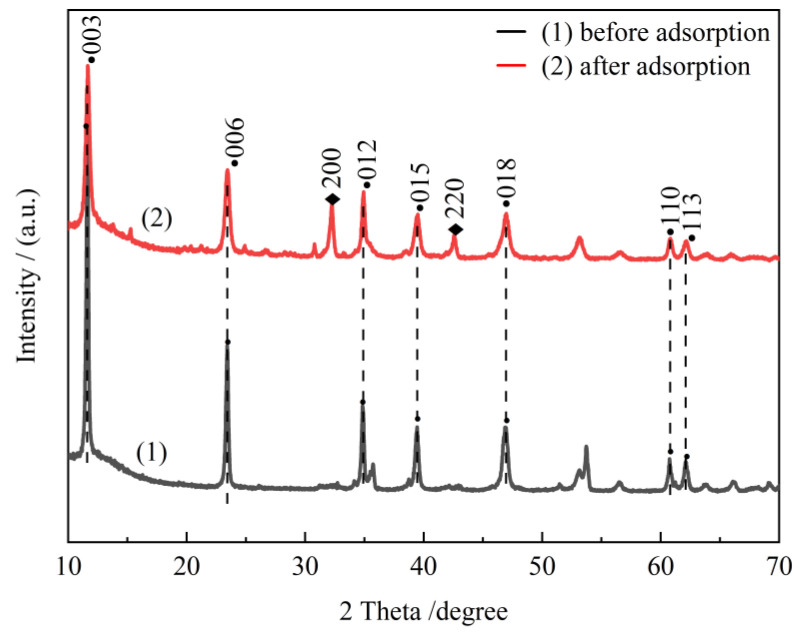
The XRD patterns of CTAC-MgAl-LDH before and after adsorption.

Figure 13 shows the XRD plots of CTAC-MgAl-LDH before and after adsorption, from which it can be seen that the adsorbent still has the characteristic crystalline of MgAl-LDH after the adsorption of organic pollutants. Furthermore, since the regeneration of the adsorbent required desorption under alkaline conditions, the diffraction peaks at 32.20° and 43.40° were assigned to the (200) and (220) crystal planes of NaCl generated by the combination of Na^+^ in NaOH and Cl^−^ in CTAC. It can be inferred that the adsorption process is mainly the anion exchange between the hydrotalcite layers. It can also be seen that the adsorption of organic matter between the hydrotalcite layers during the adsorption process destroys its ordered lamellar structure to a certain extent, which leads to a decrease in the intensity of the hydrotalcite characteristic diffraction peaks.

**Figure 14:**
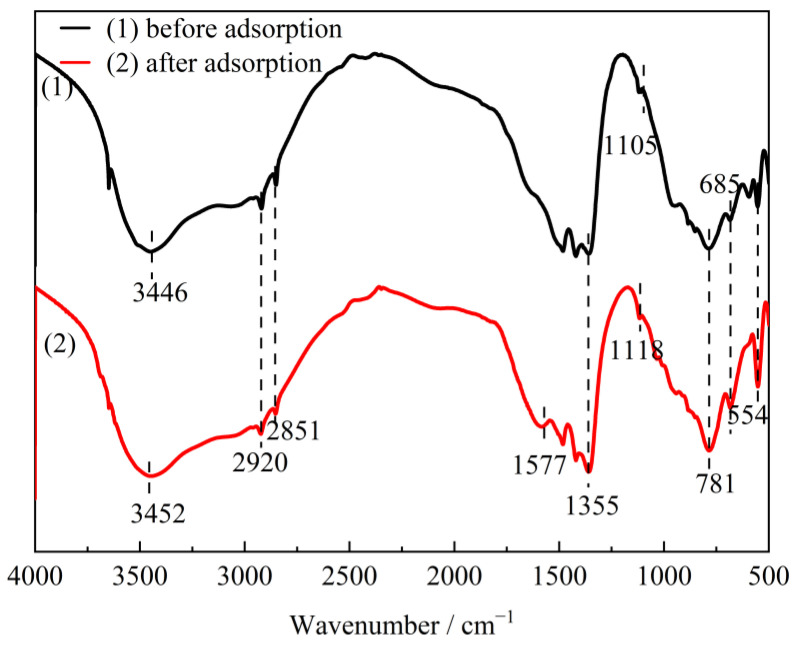
The FTIR spectra of CTAC-MgAl-LDH before and after adsorption.

CTAC-MgAl-LDH after adsorption of SL was characterized by FTIR (Figure 14) measurements. It can be seen that the similarity of frequency bands before and after adsorption indicated that the functional groups such as CH_3_ asymmetry, CH_2_-symmetric stretching vibration, and metal–oxygen and metal–hydroxy vibration of the adsorbed material were well maintained. Compared with CTAC-MgAl-LDH, after interaction with water containing SL, the sample showed an asymmetric stretching vibration of O=S=O at 1118 cm^−1^ and a C=C stretching vibration from a benzene ring skeleton in aromatic conditions at 1577 cm^−1^, indicating that the SL was adsorbed on the surface of CTAC-MgAl-LDH. In addition, the stretching vibration band of O-H shifts from 3446 cm^−1^ to 3452 cm^−1^, which is associated with the coordination bond formed by hydroxyl and carboxyl groups in sulfonated lignite and hydroxyl groups in CTAC-MgAl-LDH [49]. Since regeneration of the adsorbent required desorption under alkaline conditions, the diffraction peaks at 32.2° and 43.4° were assigned to the (200) and (220) crystal planes of NaCl generated by the combination of Na^+^ in NaOH and Cl^−^ in CTAC.

**Figure 15:**
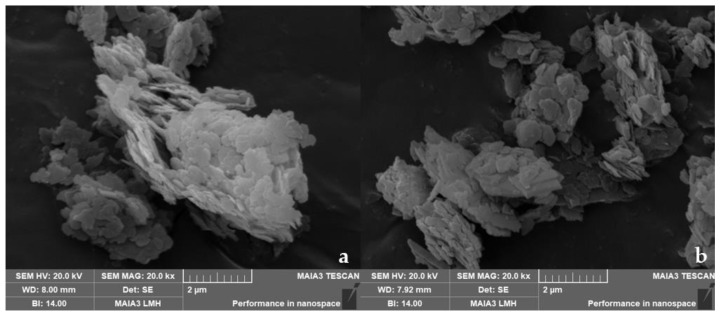
SEM image of template hydrotalcite before (**a**) and after adsorption (**b**).

Figure 15 shows scanning electron micrographs of porous hydrotalcite (CTAC-MgAl-LDH) prepared under hydrothermal conditions before and after adsorption. As can be seen from Figure 15a, the CTAC-MgAl-LDH before adsorption not only has the hexagonal lamellar structure of conventional hydrotalcite but also has a three-dimensional flower-like structure formed in the presence of surfactants. The hexagonal morphology of LDH still remained after adsorption (Figure 15b) and indeed even agglomeration occurred to some extent, which indicates that the layered structure is destroyed.

### 2.8. Adsorption Mechanism of Hierarchical MgAl-LDH

**Figure 16:**
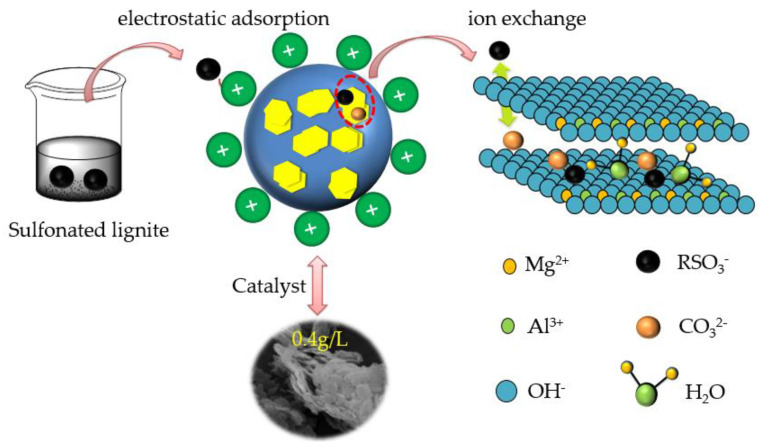
Schematic diagram of the mechanism of sulfonated lignite adsorption by CTAC-MgAl-LDH.

Based on experimental and characterization results, both physisorption and chemisorption existed in the SL adsorption of CTAC-MgAl-LDH. The initial stage of adsorption is dominated by physical adsorption while chemisorption plays a dominant role in the middle and late stages of adsorption. The sulfonated lignite molecules decompose into sulfonate ions (R-SO_3_^−^) after dissolving in the solution, as shown in Figure 16. Then, R-SO_3_^−^ were attracted by cations (Mg^2+^, Al^3+^) in the interlayer CTAC-MgAl-LDH due to the electrostatic force. Subsequently, ion exchange occurs between R-SO_3_^−^ adsorbed on the surface of CTAC-MgAl-LDH and CO_3_^2−^ in LDH layers, which effectively reduces the remaining concentration of sulfonated lignite in the water column. This is consistent with the results of adsorption kinetic studies and adsorption isotherm studies [50].

## 3. Materials and Methods

### 3.1. Materials

All chemicals including trimethyl tetradecyl ammonium chloride (TTAC), cetyl trimethyl ammonium chloride (CTAC), octadecyl trimethyl ammonium chloride (OTAC), salicylic acid (SA), Mg (NO_3_)_2_·6H_2_O, Al (NO_3_)_3_·9H_2_O, urea, sodium hydroxide (NaOH), hydrochloric acid (HCl), and absolute ethanol were of analytical grade without any further purification and supplied from Xi’an Chemical Reagent Factory (Xi’an, China). Sulfonated lignite was purchased from Tarim, Xinjiang, China. In addition, deionized water was used to formulate the solution.

### 3.2. Preparation of Porous MgAl-LDH

Hierarchical porous MgAl-LDH was prepared by hydrothermal method using micelles as soft templates. Firstly, 0.69 g of salicylic acid and 3.64 g of CTAC were dispersed into 100 mL of distilled water and stirred at 60 °C for 60 min to obtain a micelle solution. Then, Mg (NO_3_)_2_·6H_2_O and Al (NO_3_)_3_·9H_2_O with a molar ratio of 3:1:10 were dissolved in 100 mL of distilled water to obtain a metal salt solution. Subsequently, the metal salt solution was slowly added into the micelle under stirring to ensure they were fully mixed. Next, the formed suspension was transferred to an autoclave and the sealed container was then placed in the roller heating furnace at 160 °C for 6 h. Finally, the obtained precipitate was washed with deionized water and ethanol by centrifugation until the solution reached a pH of 7 and subsequently dried at 80 °C overnight. The obtained sample was labeled as CTAC-MgAl-LDH and the porous hydrotalcites with surfactants TTAC and OTAC were prepared by the same method as TTAC-MgAl-LDH and OTAC-MgAl-LDH, respectively. For comparison, the traditional MgAl-LDH in the absence of surfactant solution was prepared and designated as LDH.

### 3.3. Characterization of Materials

The phase structures of samples were characterized by an X-ray diffractometer (JDX-3530, Tokyo, Japan) with Cu Kα radiation and a scanning speed of 2 ° min^−1^ at a 40 kV voltage and a 40 mA current. All IR measurements were performed on a Nicolet 5700 FTIR spectrometer (Thermo Electron Co., Waltham, MA, USA) at room temperature in the region of 4000–500 cm^−1^. The surface area and pore structure were calculated using the BET method and the Barrett–Joyner–Halenda (BJH) model, respectively. Thermogravimetric (TG) analysis was investigated using a TGA/SDTA 851 thermal analyzer from 25 °C to 800 °C under an inert nitrogen atmosphere at a heating ratio of 10 °C·min^−1^. Scanning electron microscopy (SEM) images were captured using a field-emission scanning electron microscope (JSM-6390A).

### 3.4. Adsorption Experiments

In this typical adsorption experiment process, 0.04 g of adsorbent was added to 100 mL of a sulfonated lignite solution with an initial concentration of 100 mg/L. The mixtures were placed in a magnetic stirrer at room temperature and the sulfonated lignite concentration was determined by UV–vis spectrophotometry at the wavelength maximum absorbance of 300 nm. In the adsorption kinetics study, 0.04 g of adsorbent was added to a 250 mL beaker containing 100 mL sulfonated lignite solutions with 100 and 200 mg/L concentrations, respectively. In the adsorption isotherm experiment, 0.08 g of adsorbent was added to 50 mL of sulfonated lignite solutions with concentrations of 100, 200, 300, 400, and 500 mg/L, respectively. Adsorption thermodynamic analysis was carried out by adding 0.08 g of adsorbent to 50 mL of a sulfonated lignite solution (200 mg/L) at 298.15 and 303.15 K. The adsorption capacity (*q_t_*) at any given time and at equilibrium was calculated according to the following equation:

(8) $$q_{t}=\frac{\left(C_{0}-C_{t}\right)\times V}{m}$$

where *C*_0_ (mg/L) and *C_t_* (mg/L) are the initial and equilibrium concentrations (mg/L) of sulfonated lignite, respectively; *V* (L) is the volume of solution; and *m* (g) is the mass of the adsorbent.

## 4. Conclusions

In summary, LDH, TTAC-MgAl-LDH, CTAC-MgAl-LDH, and OTAC-MgAl-LDH were synthesized through a hydrothermal method using micelles as templates. The samples showed the best removal efficiency of SL at a carbon chain length of 16 (CTAC-MgAl-LDH) with adsorption capacities of 231.70 mg/g. The lower removal rate for samples with a carbon chain length less than 16 (LDH, TTAC-MgAl-LDH) was due to the inability of the micellar template and the salt solution to form an optimal three-dimensional network structure. For carbon chain lengths greater than 16 (OTAC-MgAl-LDH), excessive micelle viscosity makes the prepared samples poorly dispersed, which in turn affects their adsorption performance. The as-obtained CTAC-MgAl-LDH gives a very high surface area, porous hierarchical structure, and excellent dispersity, resulting in promoting the efficient exchange of interlayer anions of CTAC-MgAl-LDH with R-SO_3_^−^ of SL. Research on adsorption kinetics and isotherms indicated that the adsorption process followed pseudo-second-order kinetics and Fredich isotherm models, respectively, and therefore belongs to multi-layer chemisorption. Consequently, CTAC-MgAl-LDH exhibits superior adsorbability and excellent reusability in the adsorption of SL macromolecules. It is expected that the porous hierarchical CTAC-MgAl-LDH synthesized in this work is promising in the treatment of oilfield pollutants.

## Figures and Tables

**Table 1:** BET surface area, pore volume, and pore diameter of LDH.

Sample	The Specific Area/m^2^·g^−1^	Pore Volume/cm^3^·g^−1^	Pore Diameter/nm
LDH	19.21	0.12	2.42
TTAC-MgAl-LDH	134.55	0.19	2.16
CTAC-MgAl-LDH	174.24	0.20	4.72
OTAC-MgAl-LDH	112.81	0.19	1.63

**Table 2:** Parameters of the four dynamics models.

Kinetic Model	Parameter	Concentration/mg·L^−1^	
		100	200
Pseudo-first-order	*q_e,cal_*/mg/g model	98.87	618.19
	*k*_1_ h^−1^	0.02	0.02
	*R* ^2^	0.906	0.917
Pseudo-second-order	*q_e,cal_*/mg/g model	240.38	473.93
	*q_e,exp_*/mg/g experimol/Lent	222.46	438.77
	*k*_2_/g/m·gh	0.000022	0.00007
	*R* ^2^	0.996	0.998
Intra particle diffusion	*k_i_*_1_/mg/gh^1/2^	21.02	9.92
	*R*_1_^2^	0.998	0.906
	*k_i_*_2_/mg/gh^1/2^	0.42	3.53
	*R*_2_^2^	0.860	0.917
Liquid film diffusion	*K_fd_*/h^−1^	0.021	0.02
	*R^2^*	0.906	0.901

**Table 3:** Isothermal model parameters of SL adsorption by CTAC-MgAl-LDH.

Models	Parameter	Temperature	
		298.15 K	303.15 K
Langmuir	*q_m_*/mg/g	3839.51	2368.04
	*b*/mg/L	47,209.02	34,439.21
	*R* ^2^	0.879	0.952
Freundlich	*K_f_*/mg/g	14.18	18.30
	*n*	1.08	1.14
	*R* ^2^	0.998	0.998
D-R	*q_m_*/mg/g	403.97	396.47
	*β*/mol^2^/kJ^2^	17.26	10.42
	*R* ^2^	0.870	0.879
	*E*/J/mol	170.20	219.00

**Table 4:** Thermodynamic parameters for the adsorption of SL by CTAC-MgAl-LDH.

*T*/K	Δ*S*/J/mol·k	Δ*H*/kJ/mol	Δ*G*/kJ/mol	*R* ^2^
298.15	66.68	13.71	−6.14	0.994
303.15			−6.55	
318.15			−7.49	

## References

[1] Gao J., Feng M., Yan Y., Zhao Z., Wang Y. (2023). Preparation of a sulfonated coal@ZVI@chitosan-acrylic acid composite and study of its removal of groundwater Cr(VI). Environ. Sci. Pollut. Res. Int..

[2] Li Z., Zhang J., Qu C., Tang Y., Slaný M. (2021). Synthesis of Mg-Al hydrotalcite clay with high adsorption capacity. Materials.

[3] Ilg M., Plank J. (2016). A novel kind of concrete superplasticizer based on lignite graft copolymers. Cem. Concr. Res..

[4] Zhang R., Wang B., Ma H. (2010). Studies on Chromium (VI) adsorption on sulfonated lignite. Desalination.

[5] Cui Y., Jiao F., Wei Q., Wang X., Dong L. (2020). Flotation separation of fluorite from calcite using sulfonated lignite as depressant. Sep. Purif. Technol..

[6] Leone V., Iovino P., Capasso S., Trifuoggi M., Musmarra D. (2018). Sorption of benzene derivatives onto insolubilized humic acids. Chem. Pap..

[7] Li Y. (2020). Research Progress of Humic Acid Fertilizer on the Soil. J. Phys. Conf. Ser..

[8] Moriguchi T., Yano K., Tahara M., Yaguchi K. (2005). Metal-modified silica adsorbents for removal of humic substances in water. J. Colloid Interface Sci..

[9] Conte P., Agretto A., Spaccini R., Piccolo A. (2005). Soil remediation: Humic acids as natural surfactants in the washings of highly contaminated soils. Environ. Pollut..

[10] Zhou L., Slaný M., Bai B., Du W., Qu C., Zhang J., Tang Y. (2021). Enhanced removal of sulfonated lignite from oil wastewater with multidimensional MgAl-LDH nanoparticles. Nanomaterials.

[11] Li Y., Bai Q., Li Q., Huang H., Ni W., Wang Q., Xin X., Zhao B., Chen G. (2023). Preparation of multifunctional surfactants derived from sodium dodecylbenzene sulfonate and theiruse in oil-field chemistry. Molecules.

[12] Zhou L., Xu Z., Zhang J., Zhang Z., Tang Y. (2020). Degradation of hydroxypropyl guar gum at wide pH range by a heterogeneous Fenton-like process using bentonite-supported Cu(0). Water Sci. Technol..

[13] Li Y., Liu J., Li W., Dou M., Ma L., Wang Q., Zhao B., Chen G. (2023). Enhanced sorption for the oil spills by SDS-modified rice straw. Gels.

[14] Xie F., Gao Y., Zhang J., Bai H., Zhang J., Li Z., Zhu W. (2022). A novel bifunctional cathode for the generation and activation of H_2_O_2_ in electro-Fenton: Characteristics and mechanism. Electrochim. Acta.

[15] Yu L., Han M., He F. (2017). A review of treating oily wastewater. Arab. J. Chem..

[16] Zhang B., Yu S., Zhu Y., Shi W., Zhang R., Li L. (2016). Application of a polytetrafluoroethylene (PTFE) flat membrane for the treatment of pre-treated ASP flooding produced water in a Daqing oilfield. RSC Adv..

[17] Qi M., Lin P., Shi Q., Bai H., Zhang H., Zhu W. (2023). A metal-organic framework (MOF) and graphene oxide (GO) based peroxymonosulfate (PMS) activator applied in pollutant removal. Process Saf. Environ. Prot..

[18] Tang Y., Zhou L., Xu Z., Zhang J., Qu C., Zhang Z. (2021). Heterogeneous degradation of oil field additives by Cu(II) complex-activated persulfate oxidation. Environ. Prog. Sustain. Energy.

[19] Xie F., Zhu W., Lin P., Zhang J., Hao Z., Zhang J., Huang T. (2022). A bimetallic (Co/Fe) modified nickel foam (NF) anode as the peroxymonosulfate (PMS) activator: Characteristics and mechanism. Sep. Purif. Technol..

[20] Xie W., Zhong L., Chen J. (2007). Treatment of slightly polluted wastewater in an oil refinery using a biological aerated filter process. Wuhan Univ. J. Nat. Sci..

[21] Yang W., Kim Y., Liu P.K.T., Sahimi M., Tsotsis T.T. (2002). A study by in situ techniques of the thermal evolution of the structure of a Mg–Al–CO_3_ layered double hydroxide. Chem. Eng. Sci..

[22] Cuautli C., Ireta J. (2015). Theoretical investigations on the layer-anion interaction in Mg-Al layered double hydroxides: Influence of the anion nature and layer composition. J. Chem. Phys..

[23] Baskaran T., Christopher J., Sakthivel A. (2015). Progress on layered hydrotalcite (HT) materials as potential support and catalytic materials. RSC Adv..

[24] Yao W., Yu S., Wang J., Zou Y., Lu S., Ai Y., Alharbi N.S., Alsaedi A., Hayat T., Wang X. (2017). Enhanced removal of methyl orange on calcined glycerol-modified nanocrystallined Mg/Al layered double hydroxides. Chem. Eng. J..

[25] Gao Y., Zhu W., Liu J., Lin P., Zhang J., Huang T., Liu K. (2021). Mesoporous sulfur-doped CoFe_2_O_4_ as a new Fenton catalyst for the highly efficient pollutants removal. Appl. Catal. B Environ..

[26] Liu Y., Goebl J., Yin Y. (2013). Templated synthesis of nanostructured materials. Chem. Soc. Rev..

[27] Shi Q., Wang W., Zhang H., Bai H., Liu K., Zhang J., Li Z., Zhu W. (2023). Porous biochar derived from walnut shell as an efficient adsorbent for tetracycline removal. Bioresour. Technol..

[28] Han S., Wang Z., Meng L., Jiang N. (2016). Synthesis of uniform mesoporous ZSM-5 using hydrophilic carbon as a hard template. Mater. Chem. Phys..

[29] Xu H., Wang W. (2007). Template synthesis of multishelled Cu_2_O hollow spheres with a single-crystalline shell wall. Angew. Chem. Int. Ed. Engl..

[30] Iwase H., Kawai R., Morishima K., Takata S.-I., Yoshimura T., Shibayama M. (2019). Rheo-SANS study on relationship between micellar structures and rheological behavior of cationic gemini surfactants in solution. J. Colloid Interface Sci..

[31] Braghiroli F.L., Fierro V., Parmentier J., Pasc A., Celzard A. (2016). Easy and eco-friendly synthesis of ordered mesoporous carbons by self-assembly of tannin with a block copolymer. Green Chem..

[32] Cai P., Zheng H., Wang C., Ma H., Hu J., Pu Y., Liang P. (2012). Competitive adsorption characteristics of fluoride and phosphate on calcined Mg–Al–CO_3_ layered double hydroxides. J. Hazard. Mater..

[33] Zhang C., Shao M., Zhou L., Li Z., Xiao K., Wei M. (2016). Hierarchical NiFe layered double hydroxide hollow microspheres with highly-efficient behavior toward oxygen evolution reaction. ACS Appl. Mater. Interfaces.

[34] Nie J., Ke Y., Zheng H., Yi Y., Qin Q., Pan F., Dong P. (2012). Preparation and characterization of organo montmorillonite modified by a novel gemini surfactant. Integr. Ferroelectr..

[35] Wu Y., Qi H., Li B., Zhanhua H., Li W., Liu S. (2017). Novel hydrophobic cotton fibers adsorbent for the removal of nitrobenzene in aqueous solution. Carbohydr. Polym..

[36] Yu X.-Y., Luo T., Jia Y., Xu R.-X., Gao C., Zhang Y.-X., Liu J.-H., Huang X.-J. (2012). Three-dimensional hierarchical flower-like Mg–Al-layered double hydroxides: Highly efficient adsorbents for As(v) and Cr(vi) removal. Nanoscale.

[37] Chu H., Wang Z., Zhang Y., Wang F., Ju S., Wang L., Wang D. (2020). Using graphene sulfonate nanosheets to improve the properties of siliceous sacrificial materials: An experimental and molecular dynamics study. Materials.

[38] Wang Y., Zhao L., Hou J., Peng H., Wu J., Liu Z., Guo X. (2018). Kinetic, isotherm, and thermodynamic studies of the adsorption of dyes from aqueous solution by cellulose-based adsorbents. Water Sci. Technol..

[39] Duan N., Li Q., Liu J., Xiao H. (2015). Enhanced adsorption performance of CuO-Al_2_O_3_ composite derived from cotton template. Can. J. Chem. Eng..

[40] Yuan D., Zhou L., Fu D. (2017). Adsorption of methyl orange from aqueous solutions by calcined ZnMgAl hydrotalcite. Appl. Phys. A.

[41] Yang S., Huang G., An C., Li H., Shi Y. (2015). Adsorption behaviours of sulfonated humic acid at fly ash-water interface: Investigation of equilibrium and kinetic characteristics. Can. J. Chem. Eng..

[42] Yang X., Liu H., Han F., Jiang S., Liu L., Xia Z. (2017). Fabrication of cellulose nanocrystal from Carex meyeriana Kunth and its application in the adsorption of methylene blue. Carbohydr. Polym..

[43] Jin L., Sun Q., Xu Q., Xu Y. (2015). Adsorptive removal of anionic dyes from aqueous solutions using microgel based on nanocellulose and polyvinylamine. Bioresour. Technol..

[44] Wang N., Yang L.Y., Wang Y.G., Ouyang X.K. (2018). Fabrication of composite beads based on calcium alginate and tetraethylenepentamine-functionalized MIL-101 for adsorption of Pb(II) from aqueous solutions. Polymers.

[45] Jeppu G.P., Clement T.P. (2012). A modified Langmuir-Freundlich isotherm model for simulating pH-dependent adsorption effects. J. Contam. Hydrol..

[46] Vreysen S., Maes A. (2008). Adsorption mechanism of humic and fulvic acid onto Mg/Al layered double hydroxides. Appl. Clay Sci..

[47] Song Y., Wang S., Yang L.Y., Yu D., Wang Y.G., Ouyang X.K. (2019). Facile fabrication of core-shell/bead-like ethylenediamine-functionalized Al-pillared montmorillonite/calcium alginate for As(V) ion adsorption. Int. J. Biol. Macromol..

[48] Ye S., Jin W., Huang Q., Hu Y., Shah B.R., Li Y., Li B. (2016). Development of Mag-FMBO in clay-reinforced KGM aerogels for arsenite removal. Int. J. Biol. Macromol..

[49] Li C., Ouyang H., Tang X., Wen G., Liang A., Jiang Z. (2017). A surface enhanced Raman scattering quantitative analytical platform for detection of trace Cu coupled the catalytic reaction and gold nanoparticle aggregation with label-free Victoria blue B molecular probe. Biosens. Bioelectron..

[50] Zhao L., Li X., Quan X., Chen G. (2011). Effects of surface features on sulfur dioxide adsorption on calcined NiAl hydrotalcite-like compounds. Environ. Sci. Technol..

